# Racism in medical education and the entanglement of contents and (con-)texts: a participative reflection on teaching materials and the everyday experiences of racialized students and physicians in Germany

**DOI:** 10.3389/fpubh.2025.1436656

**Published:** 2025-02-19

**Authors:** Hans Vogt, Patricia Piberger, Felicia Boma Lazaridou

**Affiliations:** ^1^National Discrimination and Racism Monitor (NaDiRa), German Center for Integration and Migration Research (DeZIM), Berlin, Germany; ^2^Center for Research on Antisemitism, Technical University Berlin, Berlin, Germany; ^3^Department of Psychiatry and Psychotherapy, Charité University of Medicine, Berlin, Germany

**Keywords:** institutional racism, healthcare, medical education, othering, stereotyping, misrepresentation, medical habitus, Germany

## Abstract

**Background:**

Institutional racism and racial disparities in healthcare have received greater focus in the public health sciences in recent decades. The role of medical education in this context has been researched in several studies, mostly in the US, but racism in medical education remains largely underresearched in Germany. The aim of this study is to show how racist knowledge and practices exist within German medical care and are systematically transmitted in German medical education, and how this may institutionally reproduce, legitimize, reinforce, and perpetuate disadvantages.

**Methods:**

Based on consultations and preliminary interviews with civic stakeholders and experts, teaching and learning materials in German medical education were randomly sampled. These materials served as a starting point for participative reflection on racist knowledge and practices in German medical education. In the first step, the contents of teaching and learning materials were analyzed to identify terms, themes, or concepts that propagate racist ideas. Thereafter, we sought expert feedback on the analyzed content through one-on-one interviews and focus groups with physicians and medical students who self-identify as affected by racism.

**Results:**

Our study reveals two main findings. First, racist knowledge and practices are systematically transmitted and reproduced at different levels of German medical education. Second, the entanglement of multiple institutional dimensions contributes significantly to the perpetuation and legitimization of racist knowledge and practices in German medical education.

**Conclusion:**

In keeping with the state of research, the study was primarily exploratory in character and may serve as a starting point for future research on institutional racism in German healthcare and medical education. In addition to the findings that can be used to develop further research questions, initial recommendations for action by civil society, institutions, and policymakers may be derived from the interviews and focus group discussions.

## 1 Introduction

Racism is a known risk factor for health disparities, as evidenced by international studies (1, 2). Health outcomes depend on numerous factors beyond the healthcare system itself, such as social, political, biological, economic, and environmental aspects (3–6). However, there are many factors within the healthcare system, including inequitable access and discrepancies in quality of healthcare, that also influence health outcomes (7, 8). Many studies in the Western, English-speaking literature—from the U.S., the U.K., and Australia, for example—provide detailed evidence of the relationship between racism, health status, and healthcare services. Some of the key connections highlighted are the poor socio-economic conditions of racialized groups (9–13), the existence of racist bias among patients, nursing staff, medical students, and physicians (14–18), and the biologization of “race” in technology applications such as the racialized eGFR equations (19–21). In contrast, racism as a risk factor for health disparities in Germany is considerably underresearched (22–24). Namer et al. (25) outline five themes that need to be addressed to bridge this research gap. The present study concerns itself with the exploration of theme number five: “public health infrastructure, structural racism and the intersectionality of marginalization” (25). Medical education is a vital aspect of public health infrastructure, as it plays a pivotal role in the preparedness of physicians to recognize and understand social challenges, such as structural racism, and thereby contributes to the quality of healthcare (26).

Medical staff, and physicians in particular, play a central role in public healthcare and have far-reaching social competences and responsibilities. Their education can be seen as an important structural element in the societal—and especially institutional—approach to (public) health in late-modern societies and is already a central issue in the debate on racial inequalities in healthcare in the USA (17, 27). The German NKLM 2.0^1^ also includes the topic of racist discrimination in German medical education and is being critically discussed with regard to racism in the field (28, 29). Beyond the issue of racism, the section on *theory and the concept of humanity* (“Theorie und Menschenbild”) in the NKLM 2.0 states: “Healthcare and health maintenance always take place within societal, socio-economic and ideological frameworks, which doctors should critically analyze and, if necessary, help to shape”^2^ (28). Norms and patterns of reflection are interwoven with professional and academic knowledge, scientific claims, and professional practices, and shape physicians' self-images, their concepts of humanity, and their interactions with patients [cf. (30)].

A handful of studies have highlighted the medical curriculum as an area of research in the context of racism: The *Afrozensus*—the first large, systematic study to capture the realities, experiences, and perspectives of Black, African, and Afro-diasporic people in Germany—suggests that in medical education there exists a lack of engagement with othering, stereotyping, and the consequences they have for the health of those affected by anti-Black racism. In addition, the study describes the difficulties of access to the profession that are faced by Black physicians in Germany [(31), p. 139–145]. A second, more recent study on the issue of racism in German medical education focuses on the perspectives of medical students predominantly not affected by racism. While racism in medicine and healthcare is seen as a pervasive phenomenon, many students find it difficult to recognize racist behavior and structures, as there is no shared understanding of what racism is. At the same time, students expect the medical curriculum to address the racism that exists at different levels of medicine and healthcare (32). The pioneering empirical work of Hallal (33) on diversity in medical education provides some of the basic assumptions that guided our study: The unreflected internalization of “normative as well as monocultural structural features of [medical] institutions” [(33), p. 28] in Germany and the potentially consequent application of “prejudices, stereotyping, and ‘reductionist interpretations”' (33) in medical education and practice are indications that can also be derived from findings in international research and that play an important role in the present study [cf. (15, 17, 26, 34–36)].

Our study aims to show how racist knowledge and practices exist within medical care and are systematically transmitted in medical education in Germany, and how this may institutionally reproduce, legitimize, reinforce, and perpetuate disadvantages. In this paper, we present an exploratory approach to the question of institutional racism in the content of textbooks and learning apps. In addition, we explore the everyday experiences of racism in the accounts of racialized medical students and physicians as they reflect on samples of our analyzed teaching materials in one-on-one interviews and focus groups. Our research questions can be roughly summarized as follows: (1) Is racist knowledge found in medical teaching materials and practices? How is it conveyed and, if applicable, reflected on? (2) Which aspects of this knowledge are addressed by racially marginalized medical students and physicians—both with regard to their own professional positions and with regard to the influence that norms of action and biases of physicians have on patient care? (3) What role do different institutional dimensions of medical education (actors, documents, settings) play in legitimizing and perpetuating racist knowledge and practices? The results of the present study provide an initial indication of aspects and interconnections that should be considered in subsequent in-depth studies and practical discussions of teaching materials and practices in medical education. These results also contribute to the development of new indicators for the study of racism in German medical education.

## 2 Materials and methods

### 2.1 Study design: grounded theory-based triangulation

Our research design can be described as “multiple triangulation” (37, 38). The methodological process was based on the Grounded Theory approach (39). In the sense, that our recruitment, data collection, and analysis, which will be explained in Section 3.2, were iteratively connected and designed to be open-ended, so that they are oriented to continuously build on one another. The approach was adapted at every step (preliminary discussions, review of curriculum materials, interviews, focus groups). Each step was influenced by the previous one. Different materials were compared with and interlinked during the research process. Therefore, comparison played an important role both within distinct samples (coding and categorizing) as well as between various kinds of samples (triangulation). Our aim was not to create new theories on racism, but to translate empirical international research on racism in medical education into the German context. Grounded Theory was applied as a methodological principle of triangulation rather than as a method.

Triangulation confronts the challenge of complexity by integrating heterogenous perspectives; in doing so, it can open up the multiplicity of possibly contradictory perspectives, thereby strengthening the validity and reliability of the results. Regarding our study, we can speak of multiple triangulation (cf. Section 3.2), as we draw on our diversely situated perspectives as researchers as well as on a plurality of theories, data sets, and methods. Even if elements of our triangulation that are presented in the following cannot and should not be implemented completely “equally,” as suggested in the literature [(38), p. 12], this approach improves the possibility of gaining exploratory knowledge, especially with regard to interdependent institutional processes between individual, organization, and society. The present participatory analysis is an exploratory methodological-data-researcher-theoretical triangulation that aims to address, from multiple perspectives and with multiple foci, institutional racism in the everyday professional and educational life of physicians and medical students in Germany.

#### 2.1.1 Triangulation of methods, data, and observer perspectives

*Methodologically*, we combined a range of materials and data sets. As institutionally anchored forms of knowledge, teaching materials were well suited as a starting point for practice-based reflection in the interviews, which could be complemented in the focus groups via the consideration of collective perspectives on discourses and practices. Ultimately, the first step was a thematic and content analysis of texts (textbooks, learning apps, NKLM, and interview and focus group transcripts), although images (mainly photographs) in the teaching materials were also examined. This was accompanied by various elements of qualitative document analysis (teaching materials), content analysis, and documentary methods in relation to the interviews and focus groups.

*Triangulation of observer perspectives*, described by Denzin (37) as “investigator triangulation,” is closely related to methodological triangulation. Recruitment of participants, interviews, focus group discussions, and coding were conducted by the first author (HV) and the last author (FBL), two researchers with contrasting racialized and gendered social positionalities (*white*/German/man and Black/British-Nigerian/woman), and in two separate languages (German and English). This had the advantage of reaching out across broader milieus, illuminating divergent patterns of interaction (especially regarding language, racialization, and gender), and mitigating single-coder bias. These two authors also separately conducted the joint reflections on the teaching materials with the interviewees and focus group participants. The involvement of the authors in the discussion varied depending on setup and situation (one-on-one interview, focus group). The integration of feedback loops (40) with the interview and focus group participants into the data collection and analysis process helped to diversify the viewpoints and include the participants' perspectives on our interpretation. Regarding these shared spaces and feedback loops, our research method included participatory elements. The combination of the perspectives of the researchers and the participants can be understood as another form of observer triangulation. The interviewees' perspectives on the teaching materials in the context of their everyday experiences were an elementary part of our research approach. Their storytelling provides an important counter-narrative in relation to dominant (racist) narratives in medical education in Germany as it may “add necessary contextual contours to the seeming ‘objectivity' of positivist perspectives” (41).

#### 2.1.2 Theoretical triangulation: racism, knowledge, institution, medicine, and the hidden curriculum

Our *theoretical approach* was based on several main pillars and rooted in different research fields. These fields include theories that deal with racism, sociological approaches to knowledge and institutions, and research on medical knowledge and the hidden curriculum. They will not be the subject of detailed explanation here, but instead are briefly outlined as follows.

First, we referred to different definitions of or critical approaches to understanding racism in our study and emphasized different theoretical conceptions in different stages of the research process. Racialization, exclusionary practices, and differentiating power, as three main components of racism (42), were crucial for examining the sample of teaching materials and a guiding concept for the analyzing process. Racialization describes the naturalization of certain differences or characteristics by imagining them as biologically or culturally heritable; it involves the construction, categorization, homogenization and hierarchization of such groups. Exclusionary practices are concrete mechanisms for excluding members of devalued groups (43). Differentiating power is seen as an unmarked complementary norm or as a normative antithesis to the constructed racialized groups (42) and can also be understood as “whiteness” (44). Furthermore, the three components served as sensitizing concepts (45) in the interviews, where various aspects related to racism were introduced and emphasized. To fully understand and analyze the entirety of the collected material, including the interviews and focus groups, we referred to Philomena Essed's notion of “everyday racism,” which “transcends the traditional distinctions between institutional and individual racism” [(46), p. 179].^3^ Basically, we understand racism as a crucial element of global social power relations, a form of domination, and according to a widely used definition by Essed [(49), p. 448]:

***Racism*** *is about the creation of* ***hierarchies of worthiness*** *attached to groups of people identified as different in terms of (attributed) racial, or cultural (ethnic) factors. It is a historically anchored ideology, structure and process, where one racial or ethnic group privileges its members on the basis of attributed preferred values and characteristics, in order to legitimate the disadvantaging of other groups. These values and characteristics are used to assess the worthiness of* ***human beings*** *and* ***ways of being*** *in terms of related degrees of entitlement to “be”, to be validated, and to develop*.

Second, our approach is based on a sociological conceptualization of knowledge. In this conceptualization, knowledge is understood in terms of framing discourses and practices (50, 51) and includes all kinds of socially available and accepted assumptions (52). In the case of the field dealt with here, it is above all about overlaps between curricular professional knowledge—both decidedly biomedical knowledge and medical “soft skills”—and a hidden knowledge concerning habitus, practices, and norms (the “hidden curriculum,” which is described below) in medical education. Thus, the quality of the knowledge considered here results precisely from its direct involvement in institutional processes and practices: Individual attitudes merge smoothly into and eventually align with systematically imparted professional knowledge and practices or organizational structures of order and are mutually dependent on these. On the basis of this transition and the reciprocity of different forms and levels of knowledge, it becomes clear that “institution” is understood here in its immediate relationship between individuals and superordinate structures (46). According to this understanding, “institution” goes beyond organizational or legal contexts and involves the individual as a representative, target, and medium (46, 50). In this context, our research is mainly concerned with racist knowledge and practices and their circulation between different fields and levels of medical education in Germany.

Third, the function and position of medicine and the medical profession in health and society (53–56) is fundamental to our approach for two reasons: On the one hand, medical education is foundational to and a reflection of medical knowledge and practices, which in turn are essential components of (public) healthcare in late-modern societies. On the other hand, there exists a close connection between the history of medicine (as a crucial element of life sciences) and the history of racism. This entanglement of medicine and racism continues to have an impact on contemporary knowledge and practices—internationally (57), but also in Germany (58, 59). These continuities have lasting effects in several forms of institutional knowledge production. To address these in the context of our study, we referred to educational approaches regarding medical education and the hidden curriculum (30). The acquisition and application of medical knowledge goes beyond purely medical and scientific (naturalistic) knowledge and skills. Norms and patterns of action, which are often conveyed subliminally or informally via a hidden curriculum or as a medical habitus, play an essential role in medical education and in the subsequent professional claims, knowledge, and practices of physicians. Moreover, these norms and patterns of action fundamentally shape interactions between physicians and patients (26, 60, 61).

### 2.2. Recruitment, data collection, and analysis

At the start of our research, we conducted preliminary online talks with politically engaged student groups, representatives of medical associations, physicians, and researchers or research teams. These exchanges, which were documented by handwritten minutes, had the basic function of providing initial access to the field. They also served to narrow down the research field and questions, to identify problem areas, to find content-related and discursive patterns, and to sharpen analytical understanding of the different levels of institutional processes. Furthermore, these preliminary talks, especially those with medical students, served not only as a catalyst for the recruitment of interview participants, but also as an indicator for the selection of the teaching materials to be analyzed.

On the basis of the preliminary talks, we had a first look at the contents of a selection of widely used textbooks and learning apps in medical education. The sample was rather small and primarily collected using the criterion of usage distribution. It consisted of text and image materials (~100 case examples and ~800 photographs) from a handful of relevant textbooks for dermatology, for anamnesis and clinical examination, and for general and family medicine. Additionally, widely used online learning platforms (including “visual diagnosis” in various disciplines and exam questions from 2005 to 2021 in various disciplines), seminar materials, and the content of the NKLM 2.0 (28) were taken into account.^4^ Based on a content and thematic analysis, examples from our findings—corresponding to the guiding definition of racism—were summarized in a text document, which was then presented to the participants in preparation for discussion in the interviews.

The recruitment of interview and focus group participants was facilitated by the networks and references of medical associations, civil society organizations, medical students, and physicians. The participants were recruited via an open inquiry letter (in German and English) addressed to physicians and medical students who self-identify as “affected by racism” (in the German version: “selbst von Rassismus betroffen”). Interested persons could contact the responsible researchers by telephone or e-mail. The inquiry letter was distributed via various channels, such as social media, journals of state medical associations, or online newsletters of student and physician organizations (e.g., professional associations or the Association of Democratic Physicians in Germany).

Participants received compensation of €30 per interview and an additional €30 in case of participation in a focus group (see below). The interviews were conducted via the video platform Zoom. To clarify open questions and to get to know the interviewing researchers, participants were offered a preliminary interview, which in some cases was also requested. Furthermore, 18 participants were asked to fill out via e-mail a preliminary questionnaire, which was assigned to the corresponding interview transcript and finally anonymized for the analysis (for the selection of demographic distributions, see Table 1). It was made clear to the participants that completion of the questionnaire was not a condition of participation and that individual questions could also be omitted. The preliminary questionnaire asked for the following information:

*Age/Gender/Nationality/Mother tongue/Personal, family, or professional migration context/Religious affiliation or religiosity/Current occupation, household income, and highest degree/What are you studying or what have you studied? Where and when?/If already graduated: Where are you working now?*

**Table 1:** Selection of demographic distributions of interview participants.

**Number of participants**	**18**
Study context/current occupation	Medical students: 12 Practicing medical psychologist in training to become a psychotherapist: 1 Employed: 5 (as a doctor in a clinical practice: 4, as a medical expert in a public health authority: 1)
Age	23–55 years old, with most between their mid-twenties and mid-thirties
Gender	Diverse: 1 Female: 12 Male: 5
Personal, family, or professional migration context	Some with their own migration history, most with parents with a history of migration; from: Cameroon, China, East Prussia, Ethiopia, Ghana, Iran, Jordan, Mozambique, Senegal, Sri Lanka, Tanzania, Turkey, Russia, USA, Vietnam, Zambia
Religious affiliation or religiosity	Most “none,” additionally: “agnostic”/“Buddhist”/“Muslim”/“Hindu”/“unitarian universalist”/“n/a”/“Protestant”/“believer, grew up in a culturally Jewish Muslim family. Not a community member of a religious institution”/“personally no religion, Christian family”/“baptized Roman Catholic, but also grew up with Taoist/Buddhist influences”
Mother tongue	Most German, additionally: German + Arabic/English/French English/Persian/Russian/Tamil/Turkish
Interview language	German: 10 English: 8

In addition, during the interviews, participants were asked about their self-identification. Interviewees identified as, for example, a Black person, an Asian person, a Person of Color, a Woman of Color, an Afro-German, or a German. In the results section below, reference is made to the corresponding self-identification after direct quotes from the interviews.

During the interviews we first talked about personal experiences of racism in the professional and study context. The aim was to understand how racism was defined and how it was negotiated by the participants. We then came to address the content of the teaching materials and the document that was sent to the interviewees beforehand.^5^ The interviews concluded with a reflection on personal experiences in the context of the teaching materials and the document. The joint, participatory consideration of the document showed the connections between racism and different levels of the institution (e.g., regarding formal and informal teaching content) and roughly validated the researchers' assumptions that emerged from the literature analysis. The interviews were transcribed and thematically coded and analyzed using MAXQDA (62).

Based on a first thematic summary of the interview results, we created a second theme paper, which was discussed in two focus groups [with five and seven participants; for methodological aspects, see (63)].^6^ The focus group discussions were introduced by a question about the participants' perspectives on the thematic summary and then left to the group and the dynamics of the discussion. After conducting the focus groups, all data (teaching material samples and interview and focus group transcripts) were combined and subjected to a content analysis with a focus on the participants' perspectives. An initial written summary of parts of the analysis results was sent to the participants at the end to give them the opportunity to supplement or comment on the written record.

### 2.3 Ethics, informed consent, and researchers' positionality

Within the scope of the interviews and focus groups, personal data requiring special protection has been collected and processed [so-called special categories pursuant to Art. 9 (1) GDPR].^7^ Furthermore, the participants were interviewed about experiences of racism, which can have retraumatizing and triggering effects on the persons affected. Therefore, we submitted an application to the internal ethics committee in formation (EKiG) of the DeZIM Institute, which has reviewed our application as well as the attached information on the study. The research project was unanimously rated as ethically safe by two reviewing EkiG members, as long as a few recommendations were followed (ID: DI-2022-0002). Related to this, data protection information for participants and their informed consent forms regarding the collection, anonymization, and processing of their data were closely coordinated with the data protection officer of the DeZIM Institute. Written informed consent was obtained from the individuals for the publication of any potentially identifiable images or data relating to them included in this article. Furthermore, together with the informed consent form, a separate document was sent to the participants explaining the focus and method of the study. Moreover, the researchers' own roles and positionalities were critically questioned and cautiously addressed with the participants and, if requested, discussed with them in preliminary interviews.

## 3 Results

Our results uncover different levels and dimensions of the institutional space and its role in the reproduction of racist knowledge and practices. Specifically, teaching materials, as institutionally anchored documents, serve as an empirical pivotal point through which insights regarding the question of racist conditions in medical studies, and thus also in healthcare institutions, could be gained. These insights often resulted from the joint reflection on teaching materials, and specifically on their impact and interconnectedness with regard to everyday study and work experiences. Thus, on the one hand, our findings relate to content such as the use of normative racist language and the associated under-, over-, or misrepresentation of racialized groups in seminars (as reported by the participants) and photographs and case studies in textbooks and learning apps. On the other hand, we found evidence of the significant role that dispute culture, hierarchies, work contexts, and other organizational or institutional structures play in the (re-)production of racism in medical education. In this article, we focus also on the intertwining of these different dimensions and their empirical inseparability. Racism is characterized precisely by its embedding into and permeation of all levels of institutions and society (10, 46). Initially, we explore various individual results (Sections 3.1–3.3), which will be partially discussed in more detail in further articles. Subsequently, we explore their institutional entanglements (Section 3.4), which refer to the interconnections and interdependencies in which the relationships and contexts of individual agency, group formations, institutions and societal structures shape and influence one another in non-linear and unpredictable ways (64). Complexity in this sense refers to the entanglement of cultural norms, power dynamics and individual actions in shaping and being shaped by collective behavior and social outcomes.

Our empirical investigation has uncovered reciprocal relations between diverse organizational structures, speaker positions, and settings. Our “multiple triangulation” approach proved to be well suited to exploring the different levels at which racism works and the interdependencies of these levels.^8^ We found four themes that reveal essential mechanisms pertaining to racialization, exclusionary practices, and normative assumptions [cf. (42)] within the realm of medical education and healthcare. These are: (1) the systematic omission, generalization, and stereotyping of racialized patient groups, (2) the medical habitus that (re)produces an unquestioned norm of *whiteness* and neutrality in juxtaposition with the racialized “stranger,” (3) institutional structures as a mirror and catalyst for racist knowledge and practices, and (4) the entanglement of institutional levels, settings, and dimensions. These themes are outlined in the following subsections.

### 3.1 The systematic omission, generalization, and stereotyping of racialized patient groups

Several racialized groups are largely and systematically omitted from medical teaching materials and knowledge transfer practices. This omission is evident on various levels. For example, people with dark skin types are seldom mentioned in dermatological and other teaching materials, racist social conditions are hardly ever addressed as the cause and trigger of diseases, and colonial histories of medical knowledge production and practice are often ignored. The following two quotations refer to the aforementioned omission in different regards:

*Sure, the pictures used in lectures, the anatomy atlases […] there are simply white people in them, even in these drawings. So, there are not always original photos. The drawings are also “Central European standard,” so to speak. And that is worth changing*. (Physician interview 03, German-Turkish woman, hybrid bicultural identity)

*[W]e have this course called history, ethics, and law in medicine […] and they just started with the Holocaust smartly enough, because this is painful enough for German history, but when it comes to colonialism and the role of medicine in colonialism, they just didn't mention it. So, for students who are not aware, the history of German medicine starts with the Holocaust. And euthanasia and all that stuff, which is […] highly problematic, obviously, and important to talk about. But you cannot just skip stuff that happened before, which […] is still so relevant for how medicine is today, like colonialism and tropical medicine and Robert Koch and all these kinds of people, they never talked about it. So, for me, that's a kind of subtle way of producing racism by not naming, by not telling this type of story*. (Student interview 11, BIPOC)

In the rare cases where we found dark skin types in the teaching materials (books and online learning platforms), the respective patients are often described in the corresponding text material as exotic, alien/strange (e.g., “foreign”), or deviant (e.g., due to irresponsible behavior). In interviews, it is reported again and again that:

*diseases are simply not discussed with reference to dark skin, or if they are discussed with reference to dark skin, then they are just tropical diseases, so that also has something exotic about it*. (Student interview 01, Black person)

This localizes the group with the characteristic “dark skin” outside a common norm. Even beyond the illustrations—for example, in text-based case studies for exam preparation—there are descriptions whose relevance for the medical context is often unclear and that clearly operate as racial markers (see examples below). These can also lead to an internalized bias on the part of physicians when providing professional treatment:

*So really, the longer you read it, the more it seems that way. In the meantime, I also think with regard to certain population groups: Do they have a yellow fever vaccination? Where I think to myself: “so why am I suddenly thinking that? I would never have thought that before.” And then I notice, well, now that I'm preparing for my exams that many [exam] questions work very much with these stereotypes*. (Student interview 08, POC)

So, when racialized groups are represented in teaching materials or medical training, they are often located beyond supposedly Western (65) or supposedly German norms and values. Textbook examples and data from the interviews illustrate how some areas of medical studies can produce or reinforce racial bias in aspiring doctors [see also (34)]. When “non-Western” groups are vaguely generalized as “foreign,” “different,” or “particularly challenging,” they are, at the same time, stereotyped through specific attributions and often associated with certain illnesses or behaviors. In many cases, these attributions are made without sufficient empirical transparency and contextualization. For example, a book chapter from a widely used textbook on general medicine implies a potentially problematic (linguistic and content-related) separation of a generalized group of “foreigners” through its title: *Foreign Patients* (“Ausländische Patienten”). Thus, a “Tamil” or “Turkish” patient falls into the generalizing category of “foreigners” just as much as “migrants,” “asylum seekers,” “Eastern European […] men,” or “adults […] [from] sub-Saharan Africa, parts of the Caribbean [and] Thailand.” (While, for example, *white* Norwegian or Swiss foreigners do not appear in this chapter). Many of these groups are often only vaguely specified and, for example, associated with excessive “alcohol and drug consumption,” “increased risk of sexually transmitted diseases,” or “‘magical beliefs' such as voodoo, superstition, etc.” [(66), p. 179–190]. The use of the group designations decidedly associated with “ethnicity” is not explained in a consistent way (e.g., with regard to migration history, citizenship, or place of residence), nor is there any problematization of the “markers” via which these classifications could or should be made in practice [cf. (66)].

Terminology plays a fundamental role in these dynamics. Not only are biologizing, “race”-related terms such as “Caucasian” or “rassisch[…]” [(67), p. 417) still used in lectures,^9^ training courses, teaching materials, and practice. In general, terms are used inconsistently and vaguely. While the terms “Rasse” and “race” are by now less common in Germany—with the exception of references to international studies in seminars or lectures—vaguely defined categories or substitute terms such as “ethnicity,” “migration background,” or “foreigner” are frequently used. Clear examples of such substitute terms and of how they blend social and biological contexts can be seen in a textbook for anamnesis and clinical examination. While the term “Rasse” was still used in the 2018 edition [cf. (68)], it was avoided in the next edition (67). “Rasse” was replaced with “descent” [(67), p. 54], “ethnic origin” [(67), p. 74], or “skin color” [(67), p. 404]. It is only in the context of “increased body hair […]” that “racial dispositions” (“rassische Dispositionen”) are still mentioned as a factor in the new edition and distinguished from “ethnic” (“ethnische”) dispositions [(67), p. 417].

The results of our study indicate that differences in disease prevalence between different groups are often conveyed in lectures and teaching materials without sufficient context and the necessary transparency (e.g., with regard to socio-economic conditions). This can imply a subtle naturalization of certain correlations. Supposed connections between racialized characteristics and certain behaviors or disease prevalence are often presented as a given and thus possibly misrepresented and falsely internalized by the students:

*Why do you [lecturer] associate his heritage with the behavior or with an illness? […] Because the students here will associate the case with the heritage and always think: Okay, I see a person with this background, this is how I have to behave. And this is how I have to treat them, because they fall into this and that category*. (Student interview 11, BIPOC)

This kind of internalization may lead to distorted diagnoses in medical practice and may have serious consequences for patients.

### 3.2. The unquestioned norm of *whiteness* and neutrality: medical habitus and its racialized “strangers”

The systematic omission, generalization, and stereotyping of certain patient groups described above is reflected in the construction of an unquestioned norm of *whiteness* and neutrality with regard to the medical profession. In the following, we address this particular medical self-image as a medical habitus and show how it governs medical education and practice. The following passage from the abovementioned textbook chapter on GP care is an example of how teaching materials and lectures in clinical education subtly and semantically convey such a medical self-image (“us,” “own cultural area”) to medical students. An essential part of the production of such a *white* and “neutral” self-image is the juxtaposition with racially marginalized patients who are imagined as “strangers” or as the other of medical care and the medical profession. The following passage from the textbook clearly shows the othering of “people of different origins”:

*Reasons for the use of healthcare services, expectations of encounters with the doctor and the way complaints are described appear strange to us […]. Discrepancies between the patient's assessment and the doctor's assessment are often greater than with patients from the [doctor's] own cultural area*.^10^[(66), p. 190]

In many areas of medical education, a self-image of the medical profession is promoted—also by way of reference to scientific rationality and classification systems—that is decidedly distinct from the aforementioned stereotyping of and exoticizing attributions to the “other” or “stranger.” This self-image is a constitutive element of the exclusion and lack of recognition of both racialized patients and racialized medical students and physicians. It may have effects on racialized medical students and practitioners, and subsequently on the treatment of racialized patients, as will be described later in the discussion.

The *white* medical “us” is reinforced by the self-attribution and social expectation of medical-humanistic neutrality, which are thus part of the racist conditions themselves. As an ideally fundamental element of a physician's work ethic, this neutrality is repeatedly and implicitly referred to as a paradigmatic basis in various textbooks. In addition, claims of neutrality represent a barrier to a critical examination of racism because, as a socially disapproved phenomenon, racism is diametrically opposed to them. Under such conditions, the naming of racism, within institutions that are expected to meet societal standards of neutrality and objectivity, can be perceived as a more serious issue than the existence of racist structures themselves. The following quote about naming racism in lectures and seminars refers to this:

*[W]hen you somehow say: “this example is racist,” it is first of all [seen as] a much bigger problem that you even perceive it that way […] than the fact that it is actually racist and does not belong in the classroom like that*. (Student interview 10, Black cis woman)

Such defensive attitudes also persist due to a lack of clarity about what “racism” means, for example when racism is equated with right-wing extremism. Thus, the self-image of being neutral contributes to an inability to clarify these issues, because this self-image makes it difficult to address racism from the outset. The following quotes show the extent to which the claim to neutrality is linked to an internalized defensive attitude among doctors and medical students:

*Then there is this self-image: we are not discriminatory; we treat everyone the same. That definitely stands in the way of recognizing that we don't treat everyone equally, of course. I think there are, again, these fundamental processes […]. I actually think you have to question yourself more as a medical professional […]*. (Physician interview 01, POC)

*I sometimes have the feeling that people think: “Yes, I'm a medical professional and that's why I love all people and am open to everyone.” But […] they don't really want to or can't realize that although they like people and want to treat everyone equally, that's just not the case. That there are simply internalized thoughts and other feelings that we all have no influence over, simply because they are so omnipresent […]*. (Student interview 03, German-Asian woman)

Both quotes show the participants' awareness of the existing racist realities in the medical context. At the same time, the medical habitus' claim to neutrality and objectivity makes a critical examination of racism difficult, both in medical practice and in medical education.

### 3.3. Institutional structures as a mirror and catalyst for racist knowledge and practices

Medical care is often characterized by staff shortages, time constraints, heavy workloads, and rigid hierarchies. Discriminatory practices may particularly be encouraged by these care contexts that are also often characterized by incomplete information about patients (15, 23). Economic constraints and time pressures can cause or promote exclusionary patterns of action, as seen in the following example of dealing with language barriers:

*And a big problem is time. I used to work in a GP practice and each patient has ten minutes. And if a patient doesn't speak German well, then he only gets ten minutes and no extra effort is made to understand this person. It's simply that people often act on the basis of assumptions*. (Student interview 16, POC)

The challenging work contexts are linked to hierarchical structures that often represent, promote, and maintain racist conditions. The following quote illustrates how professional dependency on relationships impacts the way how racialized physicians navigate medical working spaces:

*Yes, I think this is also very pronounced in medicine, because we have a very hierarchical system, yes. There is a clear pecking order, so to speak, and then you are, especially as a student, you are at the bottom of the medical food chain, so to speak, and of course there are simply people you have to impress, yes. And then I honestly say: I do what I have to do, yes. […] You just have to get through it […]*. (Physician interview 02, Black woman)

The pronounced hierarchies and high time and performance pressures during student life are also linked to a lack of (opportunity for) reflection on study content and contexts by students, especially with regard to exchanges with lecturers. In the following quote, a student shows frustration with the refusal of lecturers to deal (self-)critically with racism:

*I sometimes find it very problematic that […] at my university, many old white men realize their problematic behavior but ignore it. […] [I]t makes me angry when people realize the problematic behavior, but then only provoke even more with their behavior by deliberately saying it anyway*. (Focus group 1, German-Asian woman)

A Black, female doctor reported that she had learned to treat racism toward patients as part of her medical practice and to intervene if necessary. However, as she explains, she tends to problematize self-experienced racism less often in order to protect physical and emotional resources—although not addressing the issue can also be burdening and stressful:

*If I approach superiors or even colleagues about it, it usually leads to nothing, yes. And ultimately it doesn't benefit me either. […] You just have to manage your resources. […] [T]he question is always, do I really take 20 minutes in my day, which is relatively stressful here anyway, to explain this to my colleague when he doesn't accept it with thanks anyway […]?* (Physician interview 02, Black woman)

It is clear here that addressing racism requires resources, which are already limited by the economic and time constraints of the medical profession. Assessing the extent to which addressing racism is expedient in terms of personal wellbeing or a change in circumstances is thus fundamental for racialized students and doctors navigating racism in the medical field.

### 3.4 The entanglement of institutional levels, settings, and dimensions

Previously indicated entanglements between levels and forms of racism in medical education are omnipresent in the data and central for the results of our study. In the following, this will be elaborated using specific examples. The underrepresentation of dark skin in dermatology books or exercise images for visual diagnosis and the misrepresentation of racialized (patient) groups in several textbooks are reflected in the underrepresentation of Black and medical students of Color. This is not only about the number of students; it is also about the ignorance of some lecturers toward a diverse student body and the de-thematization of racist and sexist discrimination. Material from a dermatology lecture provided a striking example of this: a presentation slide entitled “Which skin type am I?,” designed to encourage students to apply their new knowledge to their own bodies, shows four pictures of light-skinned people. One of the participants reported on her experience in the lecture:

*And in the dermatology lectures, for example, they also had the first four skin types according to Fitzpatrick projected on the wall. And they were just […] like […] light skin […]. And then the lecturer said: “Yes, you should somehow find yourself on these four on this slide.” And the two darkest skin types weren't on there at all. […] We make a lot of assumptions about who is in this room. And I mean, that's also in the sense that you're excluded from there*. (Student interview 06, Asian-American woman, POC)

The under- and misrepresentation of racialized people both in the teaching of medical content and in relation to the student body appears as a complementary cause and consequence of the fact that Black and students of Color are pressured to feel inferior and alone in relation to a *white* majority and norm. Due to this norm, racialized students experience being addressed and read as representatives of specific racialized groups, as singular stand-ins for collectives as a whole:

*It's just a basic feeling that you're sitting alone among almost only white people in the lecture hall or seminar. You have the feeling that you have to prove yourself more than others. You think you're being judged, so to speak, or judged more harshly […]. So, if you say things wrong, so to speak, or don't know things, they then say that this is somehow proof that you and all the other Black people don't know anything or can't do anything or are too stupid for it. So, this pressure, which isn't overt racism, but is part of everyday life, so to speak, it played a role*. (Student interview 10, Black cis woman)

This type of subtle and internalized racism—“the absence of talking about the reality of non-white people” (Student interview 11, BIPOC)—is in turn accompanied by a more obvious aspect of racism. When students, for example, call attention to the misrepresentation of certain groups in books, racism is often de-thematized through trivialization, defensive attitudes, or taboo practices and the people who are affected by racism are silenced. The *white* self-image of a neutral and objective medical profession, described above as medical habitus, reinforces these mechanisms.

The failure to recognize certain groups or individuals as medical students or doctors does not only negatively affect their identification with the medical profession. It also operates through decidedly structural (administrative) barriers that can be created and reinforced by racism, as shown by the following grave example. One interviewee reported the withdrawal of a guaranteed scholarship contract after the responsible partner hospital learned that the scholarship holder was a Black person:

*And then I was told: “Maybe you should sleep on it one more night.” And I said at that moment that it was actually quite clear to me […] that I want to accept the offer, that I want to accept it with thanks. But as far as I'm concerned, I can sleep on it for a night. Then I slept on it for a night and said: “What's up? Do we have a deal?” Then I had to send a CV, which none of my fellow students had to do, then I had to send a letter of motivation, which no one else had to do either, and then contact was suddenly broken off*. (Student interview 01, Black person)

As the person reports, the subsequent communication was dragged out by the partner hospital and the responsible study supervisors, while the study secretary told the person that racist motives obviously played a role:

*I then spoke to [a] […] student affairs [representative] […] twice a semester […] because [they] […] had told me, even when I was told about the racist motive, that I didn't have to worry—that a scholarship would be taken care of […]. That went on for a few semesters […]. And then, at the end of the sixth semester, I was told: “There were problems with your scholarship, what's going on?” [There was] a brief e-mail exchange, and then I was told: “You owe us [between* €*40,000 and* €*60,000], which we would like to have paid back in 20 days.” And that's when it actually started, that I then got legal assistance, because I was promised the whole time that a scholarship would be arranged. I also got an e-mail from [a senior representative] […] of the university saying that [they] […] would personally take care of it. […] It just got to the point where, when you've completed six semesters and are practically in your seventh semester of medicine, you don't say: “Yes, I'm going to drop out of university at this point.” Then I just had to take out the [private] loan*. (Student interview 01, Black person)

These experiences also impacted the mental health of the interviewee, who sought professional treatment to cope with the additional stress induced by racism. This connection exemplifies the entanglement and inseparability of experiences of racism on different levels that medical students and physicians may be confronted with. It is described as a chain of racism that deeply affects the overall situation of racialized students in medical education:

*I have to deal with these legal issues, which wouldn't have happened. Then there are comments from whoever. So, a comment is very obvious, but it can also be looks. When you're dealing with patients, it's things like “Where are you really from?” or […] you're somehow mistaken for the cleaning service, even though you're wearing a white coat. […] There's no way of knowing, but I'm one hundred percent sure that if this factor were removed, my grades would definitely be very different. […] That's just another part of the chain*. (Student interview 01, Black person)

## 4 Conclusion and discussion

Our study is an initial empirical approach to the interconnections between contents and (con-)texts of racial discrimination in medical education in Germany. The entanglement of norms, settings, actors, and intersectional hierarchies can be seen as a basic challenge in naming and dismantling racism in healthcare in Germany. Normative ideas of hegemonic ideals, racialization, and exclusionary practices in German healthcare concern both patients and physicians, albeit in different ways and with different intensities. It became clear in the course of our study that there are many elements that operate in connection with each other: misrepresentations of racialized patients in teaching materials; the *white* male unmarked patient; silencing of anti-racist positions in seminars as well as in administrative structures; time pressures in practice and study contexts; lack of recognition of Black and students/physicians of Color; access barriers caused by structural racism; psycho-social burdens caused by experiences of racism; rigid patriarchal hierarchies; a *white* classist medical habitus and its claims of humanist neutrality and scientific objectivity. These elements are dependent on, reinforce, and perpetuate each other.

To make our argument stronger, we focused on the omission and stereotyping of racialized patients, which was traceable through the hidden curriculum of medical education and through the self-image of the norm of whiteness and neutrality, which is bound to the medical habitus of the medical profession. One of the most important insights that was gained through the empirical material is that the exclusion of certain groups and a corresponding normativity are found at a wide variety of levels of medical education and practice. It is precisely the entanglement of different levels and dimensions that is of outstanding importance in the production, legitimization, and perpetuation of racist relations in and through medical education. As indicated in the theory section, institutional and individual bodies of knowledge work reciprocally and represent a kind of conglomerate. However, the break between the institutional mediation of a racializing *white* norm and the perspectives of the students as its “strangers” and de facto “space invaders” (69) showed how institutional structures can become visible through the individual (46). In addition, the storytelling of the racialized students and doctors as well as the joint examination of the teaching materials created a space for critical counter-narratives in order to understand, reflect on and criticize institutionalized racist narratives of the (hidden) curriculum of medical education (41). The hidden curriculum of medical education and the medical habitus were exposed as key indicators for institutional knowledge production and may be discussed in more detail in further publications.

Institutions bring stability to social power relations and negotiation processes because they change more slowly or less than the social relations that are structured by them (70). Particularly in view of this institutional inertia, it is important to examine the prevailing racist conditions, the existing misrepresentations, and the institutional structures to actively uncover the associated health and social injustices. This can only succeed if the entanglements and distortions essential to racist relations, as described in this article, are also considered. Intersectionality (71) is a relevant aspect that must be raised. Although this was not the focus, our study clearly showed that intersectionality is at work not only in the overlapping and simultaneity of different forms of discrimination, but also in the interaction and reciprocity of different levels of the institutional space. These multidimensional intersectional entanglements represent a major challenge for the analysis and must be considered in further in-depth research.

However, the professional education and socialization of doctors is far from the only reason for racism in healthcare and medicine. As was also shown in the interviews and focus groups, racism usually runs through the biographies of the participants and is experienced and perpetuated at school, among friends, in the media, in politics, and in society as a whole; sometimes, experiences in these fields operate as an antecedent to or in direct connection with experiences during student life and professional practice. Medicine and healthcare are not closed systems. While they present important, unique features as institutions with regard to social power relations or health dependencies, they have a deep impact on society and vice versa. Furthermore, grievances in healthcare also exist beyond directly racist contexts, although their consequences can be reinforced by racism. “Rationalization,” i.e. economization, bureaucratization, technologization, time pressures, and hierarchies, are problematic developments in themselves. However, in our study, they proved to be closely linked to the (re-)production and de-thematization of racist knowledge and practices.

As already indicated in the results, racism can have serious consequences for both patients and healthcare staff. Our findings support for Germany what international research has already shown for other countries. This points to various implications in practice. Some of these implications are outlined in the following. Basically, the climate of stereotyping—which is conveyed in the learning materials, courses, and practical training (con-)texts of medical education—can affect performance and be stressful and exhausting for racialized medical students (72, 73). In addition, disadvantages shown in access to study and work, as well as a lack of recognition as medical students and doctors and a resulting lack of professional identification, are connected to a lack of representation of racialized groups in the medical profession. This can have a negative impact on the quality of treatment of racialized patients (15, 31, 74).

Racist prejudices and attitudes among physicians are potentially produced, maintained, and reinforced by the normalization of racist knowledge in teaching (17, 34, 36, 75). Such a normalization was revealed in our study. Internalized attributions such as “Morbus mediterraneus,” which still occurs in medical colloquial language as a quasi-institutionalized pseudo-technical term (76), or colonialist attributions of pain perception [see (16)] play just as much of a role here as, for example, the risk of false associations between patient characteristics and the probability of illness (77) that we also find in our data. Physicians' trust in scientifically legitimized classification systems and in their own neutrality and objectivity can promote bias in decision-making, especially in contexts shaped by the institutional structures described in Section 3.3 (23). As European studies also show, racial bias among doctors can negatively influence the interpretation of legal requirements regarding access to adequate medical care [e.g., the treatment of people without health insurance in emergency situations; see (78, 79)]. Our study helps to understand how racial bias can be reinforced via medical education and training.

## 5 Limitations

Our results have various limitations, some of which are outlined here: the invitation letter or recruitment was based on self-identification as a person “affected by racism.” This could imply that participants brought a perspective critical of racism from the outset. However, this does not necessarily have to be interpreted negatively. Self-reflection on the tension between experiences of racism and the (prestigious or ambitious) social position of medical student or doctor played an important role in the interviews. In addition, the interviewees were biased with regard to the focus of the interviews because they received the sample material and topic documents before the interviews and focus groups. However, the last two aspects were consciously accepted. The aim was to undertake approaches to an underresearched field that could raise valuable questions for further research and to include perspectives of racialized individuals and groups, at least to some extent. The participants' perspectives sometimes confirmed our own initial impressions and interpretations of the teaching materials, but at other times they either negated these initial impressions and interpretations or accentuated or contextualized them differently. The participatory moment in the joint examination of the teaching materials and of the initial results of a thematic interview analysis was of central importance for the study.

## Data Availability

The datasets presented in this article are not readily available because of legal, ethical, and privacy restrictions. Requests to access the datasets should be directed to Hans Vogt, vogt@dezim-institut.de.

## Footnotes

^1^The National Competence-Based Learning Objectives Catalog for Medicine 2.0 (“Nationaler Kompetenzbasierter Lernzielkatalog Medizin”) [Medizinischer Fakultätentag (28), own translation] aims to describe the competences all medical students should acquire. It functions as a core curriculum that will lead to a new licensing regulation for physicians in Germany in 2025.

^2^Original: “Gesundheitsversorgung wie auch Gesunderhaltung ereignen sich stets innerhalb gesellschaftlicher, sozioökonomischer und weltanschaulicher Rahmenbedingungen, die von Ärztinnen und Ärzten kritisch analysiert und gegebenenfalls mitgestaltet werden sollen” (28). This and all other English translations of German-language source texts, including transcriptions of German-language interviews conducted in this study, are by the authors.

^3^For institutional racism as an analytical perspective for Germany, see Heinemann and Mecheril (47). For individual racism, see Rommelspacher (48).

^4^In the presentation of the results of our study (Section 3), this empirical material is only selectively used to demonstrate our arguments. This is primarily due to copyright issues and our decision to avoid reproducing racist images and pictures.

^5^We selected examples from the first sighting of the teaching materials described above and created a six-page document that included excerpts from the Declaration of Geneva, selected exam questions, the NKLM, and textbook case studies, as well as a description of the initial sighting results of the photographic images—everything regarding the aforementioned definition of racism. This was also the first account when the research team implicitly approached the participants with a clear definition of racism (for the second account, see text footnote ^6^). Recruitment, by contrast, was highly open and dependent on the understanding of the participants themselves (“affected by racism”).

^6^The second theme paper contained various levels that were addressed in the interviews: (1) Interpretive knowledge (definitions and norms: What is racism? What is the norm patient? What characterizes the medical profession?), (2) Process knowledge (everyday experiences: Racialized personal characteristics, othering, racialization, exclusionary practices, silencing, hierarchies, recognition as a physician, individual stress, internalization of racism), (3) Technical knowledge [medical subject and teaching content: Exclusion or stereotyping (othering) of the “non-normal”/“non-*white*,” reflection on differentiation instead of exclusion or stereotyping], (4) Challenges and opportunities, (5) Connections between the participants' own experiences of racism and the systematic unequal treatment of racialized patients.

^7^General Data Protection Regulation.

^8^The preliminary talks enabled an investigation of the various levels and nuances of possible approaches to the topic of the study. As an example, the heterogeneous perspectives on the forms or extent of racism in the medical institution or medical training can be highlighted here. The discussions ranged from an emphasis on the perspective of the affected person in everyday clinical or study life, to corresponding norms in the content of teaching materials, or rather informal contexts of academic knowledge transfer, to the problematization of concrete medical knowledge or medical “non-knowledge,” which was classified by the interlocutors as racist and scientifically or medically questionable. Linked to these different levels of racism is the question of what “institution” means in the context of the term “institutional racism.”

^9^As the German term “rassisch,” in contrast to the English “racial,” has a decidedly biological connotation (especially in the context here being discussed), it has not been translated here.

^10^Original: “Gründe für die Inanspruchnahme von Gesundheitsleistungen, Erwartungen von der Begegnung mit dem Arzt und die Art der Schilderungen von Beschwerden erscheinen uns fremdartig […]. Diskrepanzen zwischen Patienteneinschätzung und ärztlicher Bewertung sind oftmals größer als bei Patienten des eigenen Kulturkreises” [(66), p. 190].
