# Corrigendum: Racism in medical education and the entanglement of contents and (con-)texts: a participative reflection on teaching materials and the everyday experiences of racialized students and physicians in Germany

**DOI:** 10.3389/fpubh.2025.1605663

**Published:** 2025-04-14

**Authors:** Hans Vogt, Patricia Piberger, Felicia Boma Lazaridou

**Affiliations:** ^1^National Discrimination and Racism Monitor (NaDiRa), German Center for Integration and Migration Research (DeZIM), Berlin, Germany; ^2^Center for Research on Antisemitism, Technical University Berlin, Berlin, Germany; ^3^Department of Psychiatry and Psychotherapy, Charité University of Medicine, Berlin, Germany

**Keywords:** institutional racism, healthcare, medical education, othering, stereotyping, misrepresentation, medical habitus, Germany

In the published article, there was an error in the Funding statement. A specification of the funding was missed and it originally stated:

“The author(s) declare financial support was received for the research, authorship, and/or publication of this article. The study was conducted as a part of the National Discrimination and Racism Monitor (NaDiRa), which was funded by the German Federal Ministry for Family Affairs, Senior Citizens, Women and Youth (BMFSFJ).”

The correct Funding statement appears below.

